# Enhancing Working Memory Capacity in Persian Cochlear Implanted Children: A Clinical Trial Study

**Published:** 2018-03

**Authors:** Afsaneh Doosti, Maryam Jalalipour, Tayebeh Ahmadi, Seyed Basir Hashemi, Shapour Haghjou, Enayatollah Bakhshi

**Affiliations:** 1 *Department of Audiology, School of Rehabilitation Sciences, Shiraz University of Medical Sciences, Shiraz, Iran and Rehabilitation Sciences Research Center, Shiraz University of Medical Sciences, Shiraz, Iran.*; 2 *Department of Speech Therapy, School of Rehabilitation Sciences, Shiraz University of Medical Sciences, Shiraz, Iran and Rehabilitation Sciences Research Center, Shiraz University of Medical Sciences, Shiraz, Iran.*; 3 *Department of Audiology, University of Social Welfare and Rehabilitation Sciences, Tehran, Iran.*; 4 *Department of Otolaryngology, Shiraz University of Medical Sciences, Shiraz, Iran.*; 5 *Fars Cochlear Implant Center, Shiraz University of Medical Sciences, Shiraz, Iran.*; 6 *Department of Biostatistics, University of Social Welfare and Rehabilitation Sciences, Tehran, Iran.*

**Keywords:** Cochlear implant, Digit span, Non-word repetition, Working memory, Sentence repetition

## Abstract

**Introduction:**

Sensory deprivations such as hearing impairment that affect sensory input have a secondary impact on cognitive functions such as working memory (WM). WM capacity is an important cognitive component that processes language-related activities. Moreover, several studies have shown a deficit in WM in children with a cochlear implant (CI). We aimed to assess the performance of children with CIs in pre- and post-training sessions and compare their scores on a battery of WM tests to investigate the efficacy of a WM training program.

**Materials and Methods:**

Twenty-five children aged 7–10 years with a CI participated in this study. To train their WM, a computer game was used. In order to examine auditory WM, a test battery including standardized digit span (forward and backward variations), non-word and sentence repetition (subtest of the Test of Language Development–Primary) were assessed in pre- and post-training test sessions at Shiraz Implant Center.

**Results:**

There were statistically significant differences between pre- and post-training test scores on all subtests. Test score differences were statistically significant for forward digit span (P=0.003), backward digit span (P=0.001), non-word repetition (P=0.001), and sentence repetition tasks (P=0.003) before and after training sessions.

**Conclusion:**

Training may enhance WM capacity. With regards to the importance of WM in literacy and learning, it seems applying such intervention programs may be helpful in the rehabilitation of implanted children.

## Introduction

Working memory (WM) refers to the temporary storage and manipulation of information required to conduct a wide range of complex cognitive activities such as language, perception, learning, and reasoning (1-3).

WM can impact language acquisition by limiting the amount of language and relevant information that can be processed, as well as by influencing the efficacy with which new information is encoded and stored (4). Hence, its capacity is an important cognitive component to process information in language-related activities.

Baddeley and colleagues proposed one of the most widely used models of WM, the multicomponent model, which includes phonological loop, visuospatial sketchpad, episodic buffer, and central executive subsystems. The phonological loop slave system is responsible for the storage of phonological (verbal) information and prevents it being lost through consistent rehearsal. The visuospatial sketchpad maintains visual and spatial information (non-verbal) by manipulating visual pictures and developing mental strategies. The episodic buffer has limited capacity; it integrates information that comes from different sources (1,4). The central executive is also a processing system, responsible for higher order processing and controlling the phonological loop and visuospatial sketchpad subsystems (5).

Although advances in technology and intervention plans have led to improvements in the academic success of children with cochlear implants (CIs) and allow good progress in learning the spoken language, the mean performance of these children on speech and language assessments is generally below average in comparison with their peers with normal hearing. Further, as the complexity of a language skill increases, the difference between these two groups becomes more prominent (6). Similarly, the more a cognitive task relies on a phonological code, the more significant the discrepancy between implanted children and their peers becomes (6). One possible reason for this is that severe-to-profound hearing loss at an early age can cause interference in the reorganization of the prefrontal cortex and reduced maturation at the fronto-parietal regions that restrict executive functions such as planning and WM (5). Numerous studies have confirmed that WM scores are lower in implanted children when compared with normative means (5,7). The digit span test is the most widely used measure for evaluating auditory WM capacity. This test includes two variations; forward and backward digit span tests which are used to assess phonological loop and executive control systems of Baddeley's WM model (1,7-9). Researchers have shown that this measure is associated with many post implant outcome measures in children with CIs, such as speech perception, production, language development, and reading skills (1,7).

Non-word repetition (NWR), one of the most important tasks that can specifically identify phonological WM performance, requires the immediate and rapid phonological processing of a novel phonological pattern (10,11). NWR ability is highly related to language skills (i.e. vocabulary acquisition, reading and language comprehension) rather than auditory digit span tests (10,12,13). Studies have shown the significant role of the NWR task in the clinical evaluation of language acquisition in school children with CI (14,15).

Although CI provides access to spoken language, due to the significant role of the spectral structure of acoustic signal input in the formation of phonological representation, spectral degradation through CI can be partially responsible for poor performance on the NWR task in this group of children (14).

The sentence repetition task, such as the Test of Language Development–Primary (TOLD-P3), is considered a core and widely used subtest of language tests (16). In Baddeley’s 2000 revised WM model, repetition was presumed to assess the capacity of the episodic buffer, and to some degree the phonological loop (17). Studies have shown that sentence repetition is associated with WM, including phonological memory and some language skills such as grammar and vocabulary (18-21).

Generally, studies have shown that children with CI have deficits in neurocognitive processing, which is a skill necessary for language learning (22-24). It is also noteworthy that the brain is neural plastic, and imaging studies have confirmed that training programs can result in changes in the neural network and performance improvement on the trained task (25). Moreover, a recent study has indicated the necessity of implementing cognitive training in CI recipients (4,26). Hence, it is expected that interventions designed to improve WM capacity could lead to considerable gain and prevent academic complications and language delays in implanted children (14,15).

Given the importance of WM, the purpose of the current study was to evaluate the effect of a WM training program to improve WM in a sample of Persian-speaking children aged 7–10 years with CI.

## Materials and Methods

*Ethical aspects*

This study was carried out in different stages, all of which were explained to the children and their parents. The study was approved by the Ethical Committee of Shiraz University of Medical Sciences (IR.SUMS.REC.1394.194) and registered on the Iranian Registry of Clinical Trials (IRCT) website (IRCT201507 2623348N1).All participants’ parents provided signed written informed consent prior to the study.

*Participants*

In total, 41 children aged 7–10 years met the inclusion criteria at Shiraz CI center. Only 34 individuals (16 girls and 18 boys) and their parents were interested in participating in the study, of whom nine were excluded due to poor cooperation (did not attend the minimum number of sessions for WM training). Therefore, 25 children in total (11 girls and 14 boys) entered the study through convenience sampling and completed the training program. All were attending mainstream schools and had used their CI for at least 1 year. The age of implantation ranged from 17 to 60 months (mean, 32.8 ± 13.2 months). The mean age of first hearing aid fitting and CI fitting was 12.24 months (range, 7–24 months, standard deviation [SD], 4.40) and 24.40 months (range, 24.40, SD, 5.33), respectively. All participants had congenital hearing loss (20 with hereditary hearing loss while the etiology of the remaining five was unknown). Inclusion criteria were 1) Aged 7–10 years; 2) Monolingual (Persian-speaking); 3) No cerebral palsy (CP) or central auditory processing disorders (CAPD) based on their audiological report in their medical file or autism spectrum disorders (ASD) based on their medical record; 4) Normal IQ. Exclusion criteria consisted of low cooperation, defined as not attending a minimum 10 sessions of the training program (data for children who attended fewer than 10 training sessions in the clinic were not analyzed).

*Procedure*

This research was conducted in three stages at Shiraz University Implant Center, from January to March 2016. Participants who met the inclusion criteria attended the first stage (pre-test) where WM was evaluated. Then, before training, children took part in a pre-training visit to gain familiarity with the WM software. In the second stage, those who completed the minimum 10 training sessions were entered in the third stage of the study (post-test). It is worth mentioning that children were rewarded for their participation and were assessed and trained individually.

To examine whether WM training leads to improvement of auditory WM, a test battery including standardized digit span (forward and backward variations), NWR repetition and sentence repetition (subtest of TOLD) was applied in pre- and post-test sessions, and data were registered in a computer. Additionally, all test materials were read live by a female examiner (native Persian speaker) who was blind to the aims of the study. This examiner also covered her mouth with her hand to hide visual cues during examination.

*Training task*

After performing the pre-test tasks, the participants attended 16 individual WM training sessions at clinic twice a week (each training session lasted 45 minutes). The WM software comprised game-like computer-based exercises produced by Sina Cognitive Behavioral Sciences Research Institute (similar to the English version, presented by Cogmed Company), with different tasks related to WM. These tasks included auditory and visuo-spatial WM; however, based on the objective of this study, only auditory (digits and alphabet) exercises were taught during sessions. In this task, one number from 1 to 9 (auditory digit section) or one letter from the Persian alphabet (auditory alphabet section) is read aloud and the participant must select the digit or letter from among nine items presented on the screen. Digit and alphabet rehearsals (presented in forward and backward mode) started with the easy level and became more difficult with the correct response of the trainee. The program automatically increases or decreases scores depending on the individual child’s function in any given task. 

After five correct responses, the software goes to the next level with increasing number of items. If the child failed on a task, the score decreased and the child was trained at that level. In order to enhance children’s motivation to continue, they were provided with feedback through software (auditory reward) or an instructor during the training session. After completing the final training session (10–16 sessions), post-test tasks were performed.

*Pre- and post-test tasks*

*Digit span: *Forward Digit Span (FDS) and Backward Digit Span (BDS) procedures were derived from the standardized Persian version of the FDS and BDS subtests of the auditory digit span test in the Wechsler Intelligence Scale for children-Forth Edition (WISV-IV) manual. FDS is used to measure verbal rehearsal and phonological loop, while BDS is assumed to assess executive function of WM (8). Memory performance was assessed by an examiner reading aloud a series of digits.

In FDS, children were asked to repeat a series of spoken digits in the correct order. Two trials were given at each list length, beginning with a span length of two digits. In BDS, participants were instructed to repeat the digits in a reverse order. Both FDS and BDS had seven-digit sequences, each including two trial sets (trial 1, trial 2). 

If the child remembered each trial correctly, he or she would score 1, otherwise the score was 0. Therefore, the range of scoring for this test was 0–14. Digit sequences began with a span length of two digits for each trial, extending to span length of nine for FDS and eight digits for BDS. 

If a child got two lists incorrect at any given length, then testing was stopped at that point. A correct response led to a score of one 1 and an incorrect or incomplete response resulted in a score of 0. 

All scores for all trials and orders were summed and were considered the child's digit span score.

*Non-word repetition (NWR)*

The Persian version of the NWR, which includes 40 nonsense syllables in Persian, was used. The nonsense words are phoneme strings, between 1–4 syllables in length. Each child was asked to listen to one non-word presented at a rate of approximately one non-word every 3 seconds, and attempt to repeat them. If the children imitated the target non-word correctly, they would score 1, otherwise they scored 0. At the end of this task, the total possible score was 40 (11).

*Sentence repetition*

The sentence repetition subtest of TOLD consisted of 30 sentences of increasing complexity, which required the participants to repeat sentences of varying length. In the Persian version of TOLD, the minimum length of the sentences was five words; for example, sentence number 2 was “Pirâhan e jadid e man âbi ast” (“My new shirt is blue”) and sentence number 20 was “Agar shomâ khoob dars bekhânid, hatman dar emtehânât mofavvagh mishavid” (“If you study well, you certainly will pass the exams”). Children listened to sentences that were uttered live once and tried to repeat them precisely. Scoring of this test was based on the correct production of the main words in the sentences. Each sentence was scored on a 0–1 scale, with a score of 0 indicating that the child failed to repeat the sentence correctly and 1 indicating the correct repetition of the sentence. Testing was stopped when the child failed to accurately repeat five successive sentences (16).

*Data analysis*

All analyses were performed using SPSS (version 16). Due to the limited number of subjects in this study, the data did not meet normality assumptions, so nonparametric statistics (Wilcoxon test) were used to compare pre- and post-tests differences. The  level  of  significance of 0.05 with confidence intervals of 95% was chosen.

## Results

From 34 children who initially entered this study, nine were excluded since they did not attend the minimum number of sessions for WM training. Table 1 shows the mean scores, SD,the minimum and maximum scores for FDS and BDS tests, as well as the NWR and sentence repetition tests of 25 children with CIs before and after training sessions.The data demonstrate that mean scores of the test subjects improved in all of the measures(Table1).

**Table 1:** Descriptive data and test results of pre- and post-training sessions for CI children

**Subtest**	**Pre-training** **Mean (SD); min; max**	**Post-training** **Mean (SD) min**	**P-value (2-tailed/pre- & post-training)**
FDS	3.44 (0.82); 3; 6	4.00 (0.91); 3; 6	0.003
BDS	2.36 (0.70); 2; 4	3.00 (0.91); 2; 5	0.001
NWR	21.60 (5.40); 10; 30	25.64 (6.10); 11; 35	0.001
Sentence repetition	7.68(6.46); 0; 22	9.60 (7.41); 0; 25	0.003

FDS, Forward Digit Span; BDS, Backward Digit Span; NWR, Non-word Repetition

Since the data were not normally distributed, a nonparametric Wilcoxon test was used to compare the scores before and after training sessions (Table.1). The results of the Wilcoxon test show statistically significant differences between pre- and post-training test scores on all of the subtests; in other words the test score differences were statistically significant for the FDS (P=0.003), the BDS (P=0.001), the NWR (P=0.001), and the sentence repetition tasks (P=0.003) before and after training sessions.

## Discussion

The results of this study show a role for training in verbal WM improvement in Persian-speaking children with CI. A comparison of average WM tests results in 7–10 year-old CI children (our study) against normal hearing peers (11) (3.4 versus 4.9 in FDS, 2.3 versus 3.1 in BDS, 21.6 versus 35 in NWR) indicated WM difficulties in children with CI, which is in line with other studies (4,7-8,10,11,27,28). This showed the effect of auditory deprivation on cognitive components including WM which is considered the key ability in learning and language acquisition (1), producing sequential information and recalling (29). On the other hand, this supports the assumption that intervention (in the form of training) for memory and language processing skills may be effective for children with CI who have a delay in language development, speech perception and reading skills (1,7). Moreover, this finding supports the work of Bharadwaj and colleagues, who suggested that poor access to phonological structure may be responsible for such different levels of performance in children with CI (5).

The findings of the study demonstrate a significant difference between the pre- and post-test scores of WM capacity in implanted children, which shows the beneficial impact of WM training on its capacity. WM tests results (digit span, NWR, and sentence repetition) for all participants immediately after training sessions were significantly higher than those before training (Table.1).These results suggest that training of auditory skills in children can cause improvement of phonological processing skills, which is compatible with a pilot study by Kronenberger et al. (4). Their study was carried out in nine CI children, aged 7–15 years, who completed the Cogmed program (English version) over the course of 5 weeks. They concluded that some language and memory skills (sentence repetition and WM) can improve through training. It may also show an increase in activation in task-related brain areas following WM training that reflects either a more extensive recruitment of cortical areas due to an increase in the size of cortical representations or a stronger neural response in existing regions involved in the WM process (30).

Comparing digit span improvement [approximately 0.6 for FDS (3.4 to 4) and 0.7 for BDS (2.3 to 3, reach into the average norm for BDS)] indicates that this difference is not only statistically significant, but also clinically meaningful. Pisoni and colleagues showed in a large group (112 deaf and CI children who did not receive targeted WM intervention) over 8–10 years of CI use that FDS improved by only one digit and BDS had little enhancement (24). Therefore, it can be concluded that this improvement in our study over only 10–16 sessions training period is valuable. Improvement in the sentence repetition scores (a quantitative measure of children’s language skills) showed that WM improvement can be shifted towards language skills in children with CI. This suggests that the impression of training is transferable from one neurocognitive area (WM capacity) to a language processing area (sentence repetition) (4,31). Further, this finding supported studies such as one reported by Buschkuehl and colleagues which demonstrated ‘performance transfer’ of training on WM to the untrained task in children (30).

To the best of our knowledge, this is the first study evaluating the efficacy of a computer-based WM program in Persian-speaking children with CIs. In this study we evaluated the immediate effects of WM training on a test battery of some WM subcomponents and language skills. In terms of limitations of this study, we were unable to track most of the children. Also, a control group with no treatment would increase the power of the results, but the small number of children with CI in this age range at Shiraz Implant Center and a lack of consent to participate limited our study. It is recommended that future research with a larger sample size of individuals with CI, non-treatment control group, and more training sessions should be conducted in order to expand our findings. Moreover, higher order language skills including reading and writing skills, and academic performance should be evaluated. Studying cortical changes following training on WM tasks via imaging techniques is also recommended.

## Conclusion

As a general note, in this study improvements in WM capacity, NWR and sentence-repetition scores were seen in children who trained with the computer-based program. With regard to the importance of WM capacity in literacy, word learning, language processing and reading comprehension (1, 31-35), and also considering brain plastic potential, it seems that introducing such intervention programs may be helpful in the rehabilitation process of implanted children.
